# Identification and characterization of suppressor mutants of *stop1*

**DOI:** 10.1186/s12870-017-1079-2

**Published:** 2017-07-24

**Authors:** Fei Jiang, Tao Wang, Yuqi Wang, Leon V. Kochian, Fang Chen, Jiping Liu

**Affiliations:** 1 Robert W. Holley Center, US Department of Agriculture-Agricultural Research Service, Ithaca, NY 14853 USA; 2 Chengdu Institute of Biology, Chinese Academy of Sciences, Chengdu, Sichuan China; 3 College of Life Science, Sichuan University, Chengdu, Sichuan China; 4 Global Institute for Food Security, University of Saskatchewan, Saskatoon, S7N 4J8 Canada

**Keywords:** ALMT1, Aluminum toxicity, MATE, Proton toxicity, *STOP1*, Suppressor mutants

## Abstract

**Background:**

Proton stress and aluminum (Al) toxicity are major constraints limiting crop growth and yields on acid soils (pH < 5). In *Arabidopsis*, STOP1 is a master transcription factor that controls the expression of a set of well-characterized Al tolerance genes and unknown processes involved in low pH resistance. As a result, loss-of-function *stop1* mutants are extremely sensitive to low pH and Al stresses.

**Results:**

Here, we report on screens of an ethyl-methane sulphonate (EMS)-mutagenized *stop1* population and isolation of nine strong *stop1* suppressor mutants, i.e., the tolerant to proton stress (*tps*) mutants, with significantly enhanced root growth at low pH (4.3). Genetic analyses indicated these dominant and partial gain-of-function mutants are caused by mutations in single nuclear genes outside the *STOP1* locus. Physiological characterization of the responses of these *tps* mutants to excess levels of Al and other metal ions further classified them into five groups. Three *tps* mutants also displayed enhanced resistance to Al stress, indicating that these *tps* mutations partially rescue the hypersensitive phenotypes of *stop1* to both low pH stress and Al stress. The other six *tps* mutants showed enhanced resistance only to low pH stress but not to Al stress. We carried out further physiologic and mapping-by-sequencing analyses for two *tps* mutants with enhanced resistance to both low pH and Al stresses and identified the genomic regions and candidate loci in chromosomes 1 and 2 that harbor these two *TPS* genes.

**Conclusion:**

We have identified and characterized nine strong *stop1* suppressor mutants. Candidate loci for two *tps* mutations that partially rescue the hypersensitive phenotypes of *stop1* to low pH and Al stresses were identified by mapping-by-sequencing approaches. Further studies could provide insights into the structure and function of TPSs and the regulatory networks underlying the STOP1-mediated processes that lead to resistance to low pH and Al stresses in *Arabidopsis.*

## Background

Acid soils are associated with excess levels of toxic ions such as aluminum (Al^3+^), manganese (Mn^2+^), and proton (H^+^), which cause stunted growth and significant yield reductions of crops grown on acid soils [1–3]. Although applications of calcium carbonate could mitigate the acid soil associated stresses [4], these practices are expensive in financial and energy costs and, thus, are unsuitable for large scale applications, especially in developing and under-developing countries [5]. Therefore, improving crop plants’ resistance to proton and Al stresses would provide an effective solution to enhance crop yields on acid soils.

Plants have adopted two major mechanisms to cope with Al stresses, namely the Al exclusion/avoidance and the internal Al tolerance mechanisms [1, 2]. The exclusion mechanism relies on Al-activated root exudation of organic acid (OA) anions, mainly malate, citrate and oxalate, into the rhizosphere, where the OAs chelate Al^3+^ ions, forming nontoxic compounds that are unable to enter the root apex, the primary site of Al toxicity [1, 2, 6–10]. Through the internal Al tolerance mechanisms, Al retained in the root cell wall is actively removed by Al transporters, such as NRAT1 in rice [11, 12] and NIP1;2 in *Arabidopsis* [13], into the root cytosol. Then, Al in the root cell cytosol is further sequestered into root cell vacuoles and/or translocated and stored in the vacuoles of shoot cells [14–16]. In *Arabidopsis*, we have demonstrated that the NIP1;2-mediated removal of Al from the root cell wall into the root cytosol and the subsequent root-to-shoot Al translocation require a functional Al-activated and ALMT1-facilitated malate release into the root cell wall [13]. Thus, a coordinated functioning of the Al exclusion mechanism and the internal Al tolerance mechanism is required to attain overall Al tolerance in *Arabidopsis* [13].

Recently, increasing lines of evidence indicate that the root cell wall is a major target for Al toxicity [17–20], and modifications in root cell wall carbohydrate polymers (pectins and hemicelluloses), which limits binding of toxic Al^3+^ ions to the cell wall, could play an important role in Al tolerance in plants [17, 18, 21–24].

In *Arabidopsis*, *STOP1* encodes a zinc finger transcription factor that plays a critical role in plants’ resistance to proton (H^+^) and Al stresses [25]. As a result, root growth of the loss-of-function *stop1*mutants is extremely sensitive to low pH and the expression of a set of key Al tolerance genes, including *ALMT1*, *MATE*, *ALS3*, which encode an Al-activated malate transporter, an Al-activated citrate transporter and a putative transporter involved in Al redistribution, respectively, is strongly suppressed in the loss-of-function *stop1* mutant [7, 25, 26]. The fact that mutants of the key Al resistance genes, *ALMT1*, *MATE* and *ALS3*, are not hypersensitive to low pH stress indicates that the STOP1-mediated Al tolerance and low pH tolerance are independent events and tolerance to Al stress is not a prerequisite for resistance to low pH stress in *Arabidopsis* [7, 27–29]. Currently, the molecular mechanisms underlying the STOP1-mediated low pH resistance remain unknown in plants, however.

The hypersensitive phenotypes of *stop1* to low pH provide us a unique opportunity to identify *stop1* suppressor mutants with enhanced root growth under low pH conditions. Here, we report on the screens of an ethyl-methane sulphonate (EMS)-mutagenized *stop1* population and the identification of nine tolerant to proton stress (*tps*) mutants with significantly enhanced root growth at low pH. Three of the *tps* mutants also displayed increased tolerance to Al stress, two of which, i.e., *tps1* and *tps2*, were selected for further physiological characterization and mapping-by-sequencing analyses. Candidate genes and map locations were identified for these two mutants. Thus, our work could potentially open new avenues aimed at identifying previously uncharacterized genetic, cellular and regulatory components functioning in regulation of the STOP1-mediated functional networks.

## Methods

### Plant materials and growth conditions

The loss-of-function T-DNA insertion line, SALK_114180 (*stop1*), was acquired from the Arabidopsis Biological Resource Center (ABRC). Homozygous *stop1* seeds were mutagenized with EMS followed the procedures of previously reported [30]. About 500,000 M2 seeds were surface sterilized, cold-treated for 2 d, and sown onto plastic mesh floating on the Murashige and Skoog (MS) [31] solution (pH 4.3) in Magenta boxes as previously described [7, 29]. Plants were grown in a growth chamber with continuous light (130 μmol/m^2^ sec) at 23 °C. As at pH 4.3, root growth of *stop1* is severely inhibited [25], the *tps* mutants could be easily identified from the M2 population by their long-rooted phenotypes. Putative *tps* mutants were rescued from the Magenta boxes and transferred to soils. After 2 wk, young leaf tissues of individual plants were collected for genomic DNA extraction with DNeasy Plant Mini Kit (Qiagen). PCR analyses were conducted to examine the state of the original T-DNA insertions at the *STOP1* locus. The *STOP1*/T-DNA-specific primers (5′-GCTGTTGCCCGTCTCACTGGTG-3′ and 5′-GTGGTGCTCGAGAGTTCGAT-3′) were used for testing T-DNA insertions at the *STOP1* locus; the *STOP1*-specific primers (5′-GTGGTGCTCGAGAGTTCGAT-3′ and 5′-CCAACATTCCTGGGCGAGAA-3′) were used for PCR amplification of the flanking sequence encompassing the T-DNA insertion. Only those *tps* mutants that remained homozygosity of the T-DNA insertion at the *STOP1* locus were kept for further studies.

The M3 *tps* mutants were further tested for their stable long-rooted phenotypes at pH 4.3. In brief, surface-sterilized M3 seeds of individual lines were germinated on 1.2% agar plates (pH 5.6) containing 1/2 (*w*/*v*) MS salts and 1% (*w*/*v*) sucrose. Then, 4-d-old seedlings were transferred to 0.8% (*w*/*v*) gellan gum plates (pH 4.3) containing 1/2 (*w*/*v*) MS salts, 1% (*w*/*v*) sucrose. Subsequently, 5 d root growth of each seedling was measured. Seedlings with stable *tps* mutant phenotypes were transferred to soils for seed enlargements and further studies.

### Genetic analysis of the *tps* mutants

For testing dominant/recessive nature of the *tps* mutants, individual homozygous *tps* mutants were crossed with *stop1*. Surface-sterilized seeds of *stop1*, *tps*’s and their corresponding F1 progenies were germinated on gellan gum plates (pH 4.3) as described above. Root growth was measured for 5-d-old seedlings.

### Responses of *tps* mutants to excess levels of aluminum and other metal ions

To test the effects of Al toxicity, 10 ml of hydroponic solution (pH 4.3) containing 600 μM AlCl_3_ nutrients as described previously [13, 27] with a modified concentration of KH_2_PO_4_ of 0.1 mM and an addition of 1.1 mM K_2_SO_4_ was added onto the surface of gellan gum plates (pH 4.3) and dried in hood for 6 h, which resulted in final concentration of 200 μM AlCl_3_. For testing the effects of other metal ions, 1/2 (*w*/*v*) MS plates (pH 5.6) were made containing 1.2% (*w*/*v*) agar, 3% (*w*/*v*) sucrose and one of the following chemicals: 500 μM ZnSO_4_, 10 mM LiCl, 150 mM NaCl or 50 μM CdCl_2_. Then, 4-d-old seedlings were transferred from 1/2 (*w*/*v*) MS agar plates (pH 5.6) to the above mentioned treatment plates. And, 5 d root growth was measured for individual plants.

### RNA isolation and quantitative real-time qRT-PCR

About 10 mg of surface-sterilized seeds were germinated individually in Magenta boxes containing sterile hydroponic growth solution [13, 27] (pH 5.6) inside a growth chamber with a continuous light and a temperature of 23 °C. After 6 d, seedlings were transferred to fresh hydroponic growth solutions (pH 4.3) supplemented with or without 1.5 μM Al^3+^ activity for 2 d.

Total RNAs were extracted from root tissues with the RNeasy Mini Kit (Qiagen) following the manufacturer’s instruction. First-strand cDNAs were synthesized from 5 μg DNaseI-digested total RNAs using the SuperScript III First-Strand Synthesis System (Invitrogen). Real-time qRT-PCR was performed with a 7500 Fast Real-Time PCR System according to manufacturers’ protocols (Applied Biosystems, Inc.). The relative expression levels of the target genes were referred to an endogenous calibrator gene, *18S rRNA*. The sequences of the qRT-PCR primers for *ALMT1* are: CTCAGATTTTCAGATCCCAGTGGAC and TTCCCGATTCCGAGCTCATT; *MATE*: GCATAGGACTTCCGTTTGTGGCA and CGAACACAAACGCTAAGGCA; 18S: CGCTATTGGAGCTGGAATTACC and AATCCCTTAACGAGGATCCATTG.

### Detection of organic acid exudation from roots

Surface sterilized seeds (~2–3 mg) were germinated in Magenta boxes containing sterile hydroponic growth solution [13, 27] (pH 5.6) in a growth chamber with a continuous light and a temperature of 23 °C. After 6 d, seedlings were transferred to fresh hydroponic growth solutions (pH 4.3) supplemented with or without 1.5 μM Al^3+^ activity for another 2 d [27]. The exudation solutions were collected and the numbers of plants were counted at the end. Malate and citrate contents were determine by an enzymatic method described by Ryan et al., 2009 [32].

### Mapping-by-sequencing approach for identification of candidate gene regions of *TPS1* and *TPS2*

Surface-sterilized F2 seeds derived from a cross between *tps1* and *stop1* or between *tps2* and *stop1* were germinated and grown on vertical growth plates (pH 4.3) for 10 d. The *tps* mutant (long root) and non-mutant (short root) phenotypes were segregated in these F2 populations. Roughly equal amounts of leaf tissues were collected from each of ~80 long-rooted or shoot-rooted plants from corresponding F2 populations and pooled together correspondingly. Genomic DNAs were extracted from the pooled leaf samples via the E.A.N.A. Plant DNA Midi Kit (Omega Bio-tek, Inc.). Hi-Seq DNA libraries were constructed with ~2 μg DNAs via a PRC-free TruSeq prep method according to the manufacturer’s instructions (Illumina, http://illumina.com). The long-rooted and short-rooted DNA libraries were individually subjected to next generation sequencing with a High Output mode (single-end 100 bp) via a HiSeq2500 instrument (Illumina, https://illumina.com). At least 7 Gbp of sequences were generated with 50 x genome coverage for each of the libraries.

Sequencing assembly, alignments and data analyses were performed via the DNASTAR SeqMan NGen 14 software (https://www.dnastar.com). The reference genomic template, i.e., the Arabidopsis-TAIR10-dbSNP138.genome template, was downloaded from the DNASTAR SeqMan NGen 14 software for identification of non-reference SNPs/INDELs in individual DNA libraries.

## Results

### Isolation of *stop1* suppressor mutants

**Fig. 1:**
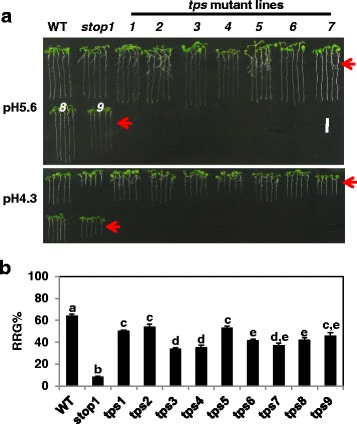
Isolation of the *stop1* suppressor mutants. Here, 4-d-old seedlings of WT (Col-0), *stop1* and the *stop1* suppressor mutants, *tps1–9*, were transferred from pH 5.6 agar plates to pH 5.6 or pH 4.3 gellan gum plates and grown vertically for 5 d. **a** Five-day root growth of individual lines at pH 5.6 (*upper panel*) and pH 4.3 (*lower panel*). **b** Relative root growth (RRG%) of individual lines. RRG% = root growth at pH 4.3/root growth at pH 5.6. Data are means ± SD (*n* = 10). Scale bar = 1 cm. *Red arrows* point to the initial root growth positions. *Letters* represent groups with significant differences (*P* ≤ 0.05) as determined by Fisher’s LSD test

At pH 5.6, root growth of the loss-of-function *Arabidopsis* T-DNA knock-out *stop1* line (SALK_114108) was comparable to that of the wild type (WT, Col-0) (Fig. 1a). However, at low pH (4.3), root growth of *stop1* was inhibited by >90%, whereas root growth of the WT was inhibited by ~35% (Fig.1a, b). These results confirmed that the *stop1* mutant is extremely hypersensitive to low pH stress [25].

We screened ~500,000 ethyl-methane sulphonate (EMS)-generated M2 seedlings with a homozygous *stop1* background and identified a total of 284 putative tolerant to proton stress (*tps*) mutants with enhanced root growth at pH 4.3. Subsequently, progenies of these putative *tps* mutants were rescreened and thirty stable *tps* mutants were confirmed. PCR analysis indicated that all of the thirty *tps* mutants retained a homozygous T-DNA insertion at the *STOP1* locus, indicating that the partially enhanced root growth phenotypes of these *tps* lines were caused by second-site gain-of-function mutations. A large portion of false putative *tps* mutants from the initial screens could be due to multiple factors, including environmental effects and high density of seedlings at the initial screen, which could jeopardize the accuracy of the initial identification of the *stop1* suppressor mutants.

Among the thirty stable *tps* mutants, nine displayed significantly enhanced root growth compared with *stop1* (Fig. 1a), whereas the rest *tps* mutants showed moderately enhanced root growth at low pH. These nine strong *tps* mutants were selected for further characterization here. Relative root growth (RRG %) (i.e., root growth at pH 4.3 vs. at pH 5.6) of these nine *tps* mutants ranged from ~35–55%, compared with the RRG%‘s of 65% and <10% for the WT and *stop1*, respectively (Fig. 1b). These results indicated that although the *tps* mutations led to significantly enhanced root growth, they could not completely recover the WT phenotype at low pH. Thus, they are partial *stop1* suppressor mutations in terms of resistance to low pH stress.

Among the nine *tps* mutants, *tps*’s *1, 2* and *5* displayed significantly higher RRG%‘s than the rest of *tps* mutants: the RRG%‘s of *tps*’s *1*, *2* and *5* were closed to or higher than 50%, whereas the RRG%‘s of the rest *tps* mutants ranged from 34 to 46% (Fig. 1). This result suggests that *tps*’s *1, 2* and *5* could be distinguished from the rest *tps* mutants.

### Genetic analysis of suppressor mutants of *stop1*

**Fig. 2:**
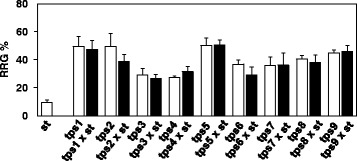
Determination of the dominant/recessive nature of the *tps* mutations. Here, 4-d-old seedlings of *st* (*stop1*), *tps*’s and their corresponding F1 progenies were transferred onto pH 5.6 or 4.3 gellan gum plates. After 5 d, root growth was measured for each seedling and RRG% was calculated for each line. RRG% = root growth at pH 4.3/root growth at pH 5.6. Values are means ± SD, *n* = 15

To test the dominant/recessive nature of the *tps* mutations, each of the nine *tps* mutants was crossed with *stop1*. At low pH (4.3), all of the F1 progenies resembled their corresponding *tps* parents when judged by their patterns of root growth, indicating that all of these *tps* mutations are dominant (Fig. 2). The F2 progenies of *tps1* x *stop1* were selected for further segregation analysis. Among the F2 progenies, the long-root and the short-root phenotypes were segregated at a ~ 3:1 ratio (Table 1). A Chi-square analysis indicated that no statistically significant difference in the expected and the observed ratio of 3:1 for long-root versus short-root phenotypes (Table 1), confirming that *tps1* is caused by a dominant mutation of a single nuclear gene.

**Table 1:** The *tps1* mutant is caused by a dominant mutation of a single nuclear gene

Cross	Observed Number of Progenies		Expected Number of Progenies			
	Suppressor Phenotype^a^	*stop1* Phenotype^b^	Suppressor Phenotype^a^	*stop1* Phenotype^b^	*χ* ^2^	*P*
*tps1* x *stop1*	166	50	183.75	61.25	3.01	0.24

^a^Long root

^b^Short root

### Responses of *tps* mutants to toxic levels of different metal ions

**Fig. 3:**
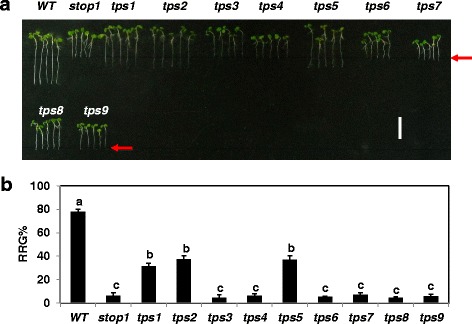
Response of *tps* mutants to Al stress. **a** Here, 4-d-old seedlings of WT, *stop1* and *tps* mutants were transferred from pH 5.6 agar plates to pH 4.3 gellan gum plates supplemented without or with 200 μM AlCl_3_. Root growth was measured for each plant 5 d after transfer. **b** RRG% of each line. RRG% = root growth (+Al) /root growth (−Al). Vertical scale bar =1 cm. *Red arrows* point to the initial root tip positions. Values are means ± SD (*n* = 10). *Letters* represent groups with significant differences (*P* ≤ 0.05) as determined by Fisher’s LSD test

Although the dominant nature makes it difficult to determine the allelic relationships between the *tps* mutants by complementation tests, their responses to treatment of different metal ions might provide clues for classification of these mutants. Therefore, we began to test the sensitivity of the *tps* mutants to Al stress. At low pH (4.3), *stop1* is extremely sensitive to Al stress: the RRG% (i.e., root growth + Al vs. root growth –Al) of *stop1* was ~6%, whereas the RRG% of the WT ~78% (Fig. 3). This result was consistent with the previously reported [25]. Although, compared with *stop1*, all of the nine strong *tps* mutants displayed significantly enhanced root growth at pH 4.3 (Fig. 1), only *tps*’s *1*, *2* and *5* showed partially, but significantly, enhanced root growth under Al stress (Fig. 3). The RRGs% of *tps*’s *1*, *2* and *5* were 4.0, 4.9 and 4.8 fold higher than the RRG% of *stop1*, but 60, 52 and 53% lower than that of WT, respectively (Fig. 3), indicating that they are partial revertant mutants of *stop1* in terms of Al resistance. As *tps*’s *1*, *2*, and *5* also displayed the highest root growth under low pH stress compared with the rest *tps* mutants (Fig. 1), they could be distinguished from the rest of the *tps* mutants.

**Fig. 4:**
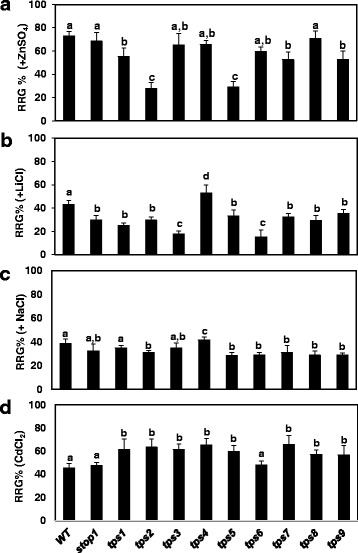
Effect of excess levels of metal ions on root growth of WT, *stop1*, and *tps* mutants. Here, 4-d-old seedlings were transferred to pH 5.6 agar plates supplemented without or with (**a**) 500 μM ZnSO_4_, (**b**) 10 mM LiCl, (**c**) 150 mM NaCl, or (**d**) 50 μM CdCl_2_. Root growth of individual seedling was measured after 5 d treatment and relative root growth (RRG%, i.e., root growth (control)/root growth (treatment) was calculated for each line. Values are mean ± SD, *n* = 15. *Letters* represent groups with significant differences (*P* < 0.01) as determined by LSD test

All *tps* mutants were further subjected to treatment with other metal ions, including Zn^2+^, Li^+^, Na^+^ and Cd^2+^. Under Zn treatment, WT and *stop1* displayed comparable root growth patterns (Fig. 4a). In contrast, *tps*’s *2* and *5* were much sensitive to Zn toxicity than the other *tps* lines which showed similar or slightly increased sensitivity to Zn toxicity compared with WT and *stop1* (Fig. 4a). As *tps*’s *1*, *2* and *5* could be grouped together based on their similar responses to low pH and Al stresses (Figs. 1, 3), the differential responses to Zn could further separate *tps1* from *tps*’s *2* and *5*.

The *stop1* mutant was more sensitive to Li stress than did WT as indicated by a 31% decrease in RRG% of *stop1* compared with that of WT under Li treatment (Fig. 4b). All *tps* mutants displayed similar sensitivity to Li stress as *stop1* did except that *tps*’s *3* and *6* were more sensitive to Li stress than *stop1*, whereas *tps4* displayed a higher level of resistance compared with WT (Fig. 4b). The WT, *stop1* and *tps* mutants manifested comparable levels of RRG% under Na stress except that *tps4* displayed slightly increased tolerant to Na stress (Fig. 4c). Surprisingly, although WT and *stop1* displayed similar sensitivity to Cd stress (Fig. 4d), all *tps* mutants, except for *tps6*, were more tolerant to Cd stress (Fig. 4d).

### Expression of key aluminum resistance genes in *tps1* and *tps2*

In *Arabidopsis*, the Al-activated and ALMT1-facilitated root malate exudation plays a major role, whereas the MATE-facilitated root citrate exudation plays a smaller but significant role, in Al resistance [7, 27]. In addition, expression of *ALMT1*, *MATE* as well as *ALS3* is controlled by STOP1 [7, 26]. Therefore, it is interesting to understand the effects of *tps* mutations on the expression of these Al resistance genes.

**Fig. 5:**
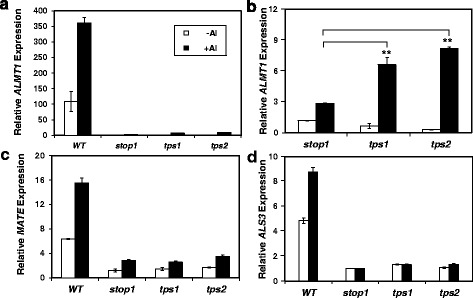
qRT-PCR analyses of expression of *ALMT1* (**a**, **b**), *MATE* (**c**) and *ALS3* (**d**) in WT, *stop1*, *tps1* and *tps2* treated without or with treatment of 1.5 μM Al^3+^ activity at pH 4.3 for 2 d. **, significant differences (*P* < 0.01) as indicated. *n* = 3

To begin with, we investigated the expression of *ALMT1*, *MATE* and *ALS3* in the root of *tps1* and *tps2*, both of which showed enhanced resistance to proton and Al stresses (Figs. 1 and 3). Real-time qRT-PCR analyses indicated that Al stress induced a strong upregulation of *ALMT1*, *MATE* and *ALS3* expression in the root of WT and the levels of the Al-activated *ALTM1* expression were much higher than those of *MATE* and *ALS3* in WT (Fig. 5a–d). These results confirmed the major role of *ALMT1* in Al resistance in *Arabidopsis* [7, 28, 29]. In addition, we confirmed that the expression of *ALMT1*, *MATE* and *ALS3* was greatly suppressed in the loss-of-function *stop1* background (Fig. 5a–d).

We notice that in *stop1*, Al treatment caused small, but significant, increases in *ALMT1* and *MATE* transcript levels, whereas *ALS3* expression was not affected by Al treatment (Fig. 5b, c and d). These results suggest that although STOP1 plays a key role in controlling the Al-induced expression of *ALMT1* and *MATE*, there exist non-STOP1 regulatory factors that control a smaller portion of Al-induced *ALMT1* and *MATE* expression, whereas the expression of *ALS3* is likely to be solely controlled by STOP1.

Compared with *stop1*, the levels of Al-induced *ALMT1* transcripts increased 1.3 and 1.9 fold in *tps1* and *tps2*, respectively (Fig. 5b), suggesting that the wild-type TPS1 and TPS2 might function as suppressors for the Al-induced and STOP1-independent *ALMT1* expression. In contrast, no significant differences were found in the patterns of *MATE* expression between *stop1*, *tps1* and *tps2* (Fig. 5c), suggesting that TPS1 and TPS2 are not involved in regulation of the Al-induced and STOP1-independent *MATE* expression.

### Root organic acid exudation in *tps1* and *tps2*

**Fig. 6:**
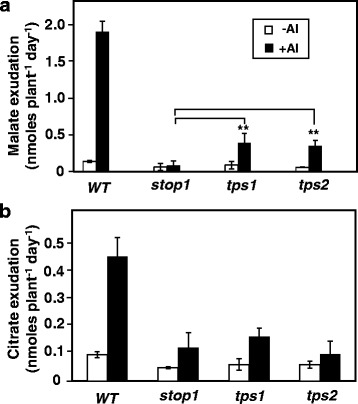
Root malate (**a**) and citrate (**b**) exudation in WT, *stop1*, *tps1* and *tps2* treated without or with 1.5 μM Al^3+^ activity at pH 4.3. **, significant differences (*P* < 0.01) as indicated. *n* = 3

Root OA exudation was measured for WT, *stop1*, *tps1* and *tps2*. In WT, Al triggered a large increase in root malate exudation and a smaller increase in root citrate exudation (Fig. 6). Compared with the WT, the Al-activated malate and citrate exudation was strongly suppressed in *stop1*: the rates of Al-activated malate and citrate exudation in *stop1* decreased by 96 and 73%, respectively (Fig. 6). These results were consistent with previously reported [7]. Interestingly, compared with *stop1*, Al treatment caused 3.6- and 3.1-fold increases in Al-activated root malate exudation in *tps1* and *tps2*, respectively (Fig. 6a). In contrast, patterns of root citrate exudation remained comparable between *stop1*, *tps1* and *tps2* (Fig. 6b).

Regression analyses indicated that levels of Al-induced *ATMT1* expression (Fig. 5) and Al-activated malate exudation were highly associated among WT, *stop1*, *tps1* and *tps2* (*R* ^2^ = 0.98). These results suggest that the increased Al resistance in *tps1* and *tps2* (Fig. 3) was due, at least partially, to enhanced Al-induced and STOP1-independent *ALMT1* expression and the associated ALMT1-mediated root malate exudation. In contrast, no correlations could be found between Al resistance (Fig. 1), *MATE* expression and root citrate exudation, suggesting that Al-induced *MATE* expression (Fig. 5c) and Al-activated root citrate exudation (Fig. 6b) had few contributions to enhanced Al resistance in *tps1* and *tps2*.

### Identification of candidate genomic regions that harbor *tps1* and *tps2* mutations by whole genome sequencing

**Fig. 7:**
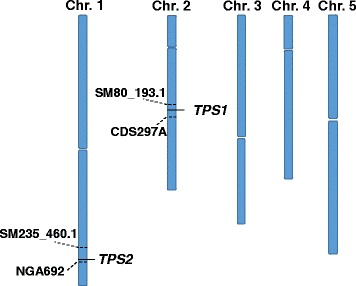
Map locations of the *TPS1* and *TPS2* regions. SM235_460.1 and NGA692, molecular markers in chromosome1; SM80_193.1 and CDS297A, molecular markers in chromosome 2

To understand the molecular bases underlying how TPSs function in the STOP-mediated signaling/regulatory networks, we started to map and clone the *TPS1* and *TPS2* loci via a mapping-by-sequencing technique. In contrast to traditional map-based cloning techniques, the mapping-by-sequencing approach is a combination of bulked segregant analysis [33, 34] and whole genome sequencing [35]. To identify candidate gene regions for *TPS1* and *TPS2*, HiSeq DNA libraries from bulked long-rooted or short-rooted F2 progenies derived from a cross between *stop1* (Col-0) and *tps1* or *tps2* were individually subjected to next generation whole genome sequencing. The sequencing data were then subjected to reference-guided assemblies and analyses with the SeqMan NGen 14 software (DNASTAR Lasergene). From each pool, non-reference SNPs/INDELs were identified and their allele frequencies calculated. As both *tps1* and *tps2* are dominant mutants, the causal non-reference SNPs/INDELs could be characterized by their allele frequencies >75% in the long-rooted mutant DNA pools, but <25% in the short-rooted non-mutant DNA pools. In addition, there would be a group of non-reference SNPs/INDELs tightly linked to the causal SNPs/INDELs with high allele frequencies in the mutant libraries due to linkage effects. On the basis of these criteria, *TPS1* was mapped to the long arm of chromosome 2 between molecular markers CDS297A and SM80_193.1, whereas *TPS2* to chromosome 1 between NGA692 and SM235_460.1 (Fig. 7). Eight and six strong candidate genes with nonsynonymous mutations were identified in the *TSP1* and *TSP2* regions, respectively (Tables 2 and 3).

**Table 2:** Candidate genes in the *TPS1* region at chromosome 2

Candidate Genes	Description	Predicted Subcellular Localization	GO Biological Process
At2g17790	Similar to yeast VPS35.	Intracellular membranes	Intracelluar protein transport
At2g27880	AGO5; required for antiviral RNA silencing	Cytosol	Defense response, Gene silencing
At2g29210	Splicing factor PWI domaincontaining protein	Nucleus	RNA splicing, mRNA processing
At2g31862	B3 domain protein	Nucleus	Regulation of transcription
At2g31890	RAP, containing putative RNA binding domain	Chloroplast, nucleus	Chloroplast rRNA processing
At2g34810	BBE16, FAD-binding Berberine family protein	Cytosol	Oxidation-reduction process, response to jasmonic acid, response to wounding
At2g43180	Phosphoenolpyruvate carboxylase family protein	Chloroplast	Catalytic activity
At2g44440	EML4, ENT domaincontaining protein	Nucleus	Defense response to fungus

**Table 3:** Candidate genes in the *TPS2* region at chromosome 1

Candidate Genes	Description	Predicted Subcellular Localization	GO Biological Process
At1g65610	KOR2	Integral component of membrane, plasma membrane	Cell wall organization
At1g71090	PIN-Likes 2	ER membrane	Auxin homeostasis
At1g72760	Protein kinase	Nucleus	Kinase activity
At1g73687	MIR159 targeting MYB family members	Cytosol	Gene silencing by miRNA
At1g74280	Hydrolases superfamily protein	Integral component of membrane	Hydrolase activity
At1g74410	RING/U-box superfamily protein	Integral component of membrane	Defense response

## Discussion

*STOP1* encodes a master transcription factor that controls both low pH and Al resistance in *Arabidopsis* [25, 26]. The fact that mutations in the STOP1-controlled Al tolerance genes, such as *ALMT1* and *MATE*, caused hyper-sensitivity to Al stress but not to low pH stress indicates that STOP1 mediates independent processes leading to resistance to low pH stress or Al stress [7]. However, the genetic and regulatory networks underlying STOP1-mediated resistance to low pH stress and Al stress remains unknown.

In this report, through classic forward genetic approaches, we have identified nine strong *stop1* suppressor mutants, which displayed partially, but significantly, enhanced root growth at low pH (Fig. 1). Genetic analyses indicated that all of the nine *tps* mutants are caused by gain-of-function dominant mutations (Table 1).

**Fig. 8:**
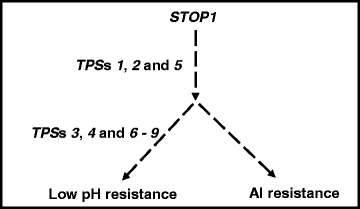
Putative *STOP1*-mediated signaling and genetic networks. *STOP1* mediates independent processes leading to resistance to low pH or Al stress. *TPS*s *1*, *2*, and *5* could be placed in the branch before the divergence of the resistance to low pH and Al stress, while *TPS*s *3*, *4*, *6–9* could be placed in the branch that is specifically involved in low pH resistance

Further physiological characterizations indicated that *tps*’s *1*, *2* and 5 also showed partially enhanced Al resistance (Fig. 3). Thus, *TPS*s *1*, *2* and *5* appear to act in a STOP1-mediated networks before the divergence of resistance to low pH and Al stresses or they promote root growth in a STOP1-independent way (Fig. 8). As the rest of *tps* mutantions caused enhanced resistance only to low pH stress (Figs. 1, 3), these mutations could be placed in the branch that is specific to low pH resistance (Fig. 8). As the effects of Al stress on plant growth can only be manifested when pH is below 5.0, low pH stress is thus always associated with Al stress. Therefore, the mutant screening strategy used in this report was not designed to identify *stop1* suppressor mutants with enhanced resistance specifically to Al stress.

Due to the dominant nature of all *tps* mutants identified here, it is hard to classify them through complementation tests. However, based on their responses to low pH and excess levels of different metal ions, these *tps* mutants could be classified into at least five different groups: Group 1 includes *tps1* which displayed significantly enhanced resistance to both low pH (Fig. 1) and Al stress (Fig. 3); Group 2 contains *tps*’s *2* and *5*, which showed similar levels of enhanced resistance to low pH and Al stresses as *tps1*, but with enhanced sensitivity to excess Zn (Fig. 4a); Group 3 includes *tps*’s *3* and *6*, which are hypersensitive to Li^+^ (a more toxic analog for Na^+^) stress (Fig. 4b). Interestingly, *tps*’s *3* and *6* did not show significant hypersensitive to Na stress compared with *stop1* (Fig. 4c). In addition, all *tps* mutants, except for *tps 6*, showed enhanced resistance to Cd stress, which might further distinguish *tps 6* from *tps 3*; Group 4 contains *tps4* which showed enhanced tolerance to Li stress (Fig. 4b); Group 5 includes *tps*’s *7*, *8* and *9*, which lack the specific characteristics of the above groups besides their increased resistance to low pH stress (Fig. 1).

It has been well characterized that one of the deleterious effects of salt (Na) stress on plant growth is the disruption of cellular K^+^ homeostasis through inhibition of K^+^ uptake that is facilitated by K^+^ channels such as AKT1 [36, 37]. Biochemical studies indicate that CIPK23 (CBL-interacting protein kinase 23) is required for activation of the AKT1 channel through the phosphorylation of the ankyrin repeat domain of the AKT1 protein [38]. Interestingly, the expression of *CIPK23* is induced by Al and low pH stresses in WT [26]. However, such an induction was strongly suppressed in the *stop1* mutant, implicating a disruption of K^+^ homeostasis in *stop1* under low pH and Al stresses [26]. This could explain the reason for the hypersensitivity of the *stop1* mutant to Na and Li stresses (Fig. 4b and c). It will be interesting to investigate if the enhanced resistance of *tps4* to Li and Na stresses is caused by up-regulation of *CIPK23* expression, whereas the enhanced sensitivity of *tps3* and *tps 5* to Li and Na is due to further down-regulation of *CIPK23* expression.

As the Al-induced *ALMT1* and *MATE* expression and the Al-activated root malate and citrate exudation are strongly suppressed in the *stop1* mutant background (Figs. 5 and 6), we decided to investigate how the *tps1* and *tps2* mutations affects *ALMT* and *MATE* expression and corresponding root OA exudation in the *stop1* mutant background. qRT-PCR analyses indicated that there exist Al-induced and STOP1-independent *ALMT1* and *MATE* expression in *Arabidopsis* (Fig. 5b and c). Interestingly, this Al-induced and STOP1-independent *ALMT1* expression was further enhanced by the *tps1* and *tps2* mutations (Fig. 5b), suggesting that TPS1 and TPS2 might function as negative regulators for the STOP1-independent *ALMT1* expression. Therefore, the increased Al resistance in *tps1* and *tps2* mutants could be due, at least partially, to the increased Al-activated, STOP1-independent and ALMT1-mediated root malate exudation. Interestingly, the Al-induced and STOP1-independent *MATE* expression was not affected by these *tps* mutations (Fig. 5c).

To identify chromosome and genomic regions for *TPS1* and *TPS2*, we carried out a mapping-by-sequencing approach to identify the candidate causal nonsynonymous SNPs in coding sequences. Compared with the traditional map-based cloning technique, which is time-consuming and labor intensive, the mapping-by-sequencing approach is a relatively simple and quick way to map and to identify the candidate causal genes. Through analyses of the distributions and the allele frequencies of the non-reference SNPs in the long-rooted and short-rooted DNA libraries, *TPS1* was mapped to chromosome 2 between the molecular markers CDS297A and SM80_193.1, whereas *TPS2* to chromosome 1 between NGA692 and SM235_460.1 (Fig. 7). Eight and six candidate genes with nonsynonymous mutations in the coding sequences were identified in the *TPS1* and *TPS2* regions, respectively (Tables 2 and 3). These candidate genes encode proteins involved in regulation of transcription, responses to hormone stimulus, gene silencing, defense responses, response to wounding and intracellular protein transport. Further functional characterization for these candidate genes will allow us to confirm the molecular identities and functions of *TPS1* and *TPS2*.

## Conclusions

We have identified nine strong *stop1* suppressor mutants, which could be classified into five groups. Two of the *tps* mutants with enhanced resistance to both low pH and Al stresses were chosen for further physiological analyses and mapping-by-sequencing gene identification procedures [39–41]. Candidate causal genes have been identified for these two mutants. Our studies represent the first steps towards the identification of the molecular identities of all *TPS* genes, which will provide insights into the structure and function of the gene products and their roles in the STOP1-mediated genetic, cellular and regulatory networks that are involved in resistance to low pH and Al stresses in *Arabidopsis*.

## Abbreviations

ALMT: Aluminum activated malate transporter
ALS: Aluminum sensitive
EMS: Ethyl-methane sulphonate
MATE: multidrug and toxic compound extrusion
MS: Murashige and Skoog
NGS: Next-generation sequencing
OA: Organic acid
RRG: Relative root growth
st: STOP1
STOP1: Sensitive to proton rhizotoxicity 1
TPS: Tolerant to proton stress
